# What is your diagnosis?

**DOI:** 10.4274/jtgga.galenos.2019.2019.0048

**Published:** 2020-03-06

**Authors:** Anupama Bahadur, Latika Chawla, Namrata Bhattacharya, Shashi Prateek, Ajay Kumar, Balram Ji Omar, Aditi Jindal, Ashok Singh, Nirali Kapoor

**Affiliations:** 1 Department of Obstetrics and Gynecology, All India Institute of Medical Sciences, New Delhi, India; 2 Department of Surgery, All India Institute of Medical Sciences, New Delhi, India; 3 Department of Microbiology, All India Institute of Medical Sciences, New Delhi, India; 4 Department of Pathology, All India Institute of Medical Sciences, New Delhi, India

A para 4, 35-year-old woman with 3 living issues and farmer by occupation presented to our hospital with symptoms of acute urinary retention for which an indwelling urinary catheter was inserted. There were no associated symptoms of hematuria, frequency, urgency or burning micturition. She also had a history of progressively increasing lump abdomen for past 6 months with dull aching pain. The patient gave a history of having undergone laparotomies twice during her childhood for a liver pathology, but she was unaware of the actual diagnosis. No records were available.

A physical examination revealed a 10x8 cm cystic abdominal mass in the left lumbar region. The mass was freely mobile and non-tender. There was another large abdomino-pelvic lump corresponding to 18-20 weeks’ gestation, cystic, mobile from side to side, non-tender, and the lower border could not be discerned. A per vaginal examination revealed a normal-sized uterus with a large 10x15 cm cystic mass felt through the right and posterior fornix.

Ultrasound revealed two large multi-septated cystic lesions on the right and left side reaching up to the epigastrium with multiple small cysts seen in both masses with no increase in vascularity, no solid areas, and no free fluid in the abdomen (Figure 1).

Contrast-enhanced magnetic resonance imaging (MRI) showed non-enhancing peritoneal and omental-based clusters of non-loculated cystic lesions in the mid abdomen (14x11x11 cm), in the left iliac fossa (6x7x7 cm), and in the pelvis (13x10x10 cm) with multiple small cysts within (Figure 2).

The patient was planned for a laparotomy. Intra-operatively, the uterus was normal size. A 10x15 cm clear cyst was seen arising from the left ovary with multiple small cysts with viscous pale, yellow fluid inside them. A similar 2x2 cm cyst was seen arising from the right ovary, and a 20x10 cm cyst and a 5x6 cm cyst were seen arising from the greater omentum (Figure 3). Total abdominal hysterectomy with bilateral salpingo-oophorectomy with omental cyst excision was performed.

## 

### Answer

A histopathologic examination revealed hydatid cysts lined by an innermost germinal layer with attached brood capsule and daughter cysts. An avascular, eosinophilic, and refractile laminated membrane was seen. The outer layer was fibrovascular with chronic inflammatory cells and giant cell reaction. To our surprise, even the myometrium had evidence of hydatid cysts (Figure 3).

The patient was a farmer and livestock handler by occupation. Ultrasound and MRI findings along with the history of two previous surgeries in the past raised a high index of suspicion for hydatid cyst. Pre-operative echinococcal serology was positive. CA-125 was normal. With a provisional diagnosis of suspected peritoneal and ovarian hydatid cyst, the patient was given a course of albendazole 400 mg twice daily for 6 weeks, following which she was taken for surgery.

Echinococcosis or hydatidosis is caused by larvae of tapeworm Echinococcus granulosus, belonging to the family Taeniidae. With dogs being the dominant carriers, Echinococcus often infects humans via echinococcus eggs in contaminated food, water or contact with infected animals (1). We present a case of a 35-year-old woman with co-existing ovarian, myometrial, and omental hydatid cysts.

Hydatid disease is an endemic parasitosis in regions such as China, Russia, South America, the Mediterranean Region, Eastern Europe, and Central Asia (2). Prevalence of hydatid disease in liver or lung is 80% (3). Very rarely, the disease has pelvic or omental involvement (4). The prevalence of pelvic hydatid cysts requiring surgery was reported as <1% (5). Due to the non-specific symptoms such as abnormal uterine bleeding, sterility and urinary retention, diagnosis and treatment of pelvic hydatid cyst poses a challenge. Cysts with unusual location such as the ovary and uterus tend to grow slowly, leaving the patient asymptomatic for a long time (6).

The World Health Organization (WHO) Informal Working Group Classification of cystic echinococcosis (CE) is shown in Table 1 (3). Diagnosis can be made by imaging, ultrasonography (USG) or computed tomography or MRI in conjunction with serology. USG lacks sensitivity for the determination of cyst viability. The sensitivity of USG for the evaluation of Echinococcus is 90% to 95% (7). Amongst the serologic tests, specific immunoglobulin G ELISA is the most sensitive measure. However, there is no consistent correlation between the number or size of cysts and serologic results (8). Serologic tests are more reliable for the diagnosis of echinococcus multilocularis infection than echinococcus granulosus infection. Antibody detection is more sensitive than antigen detection for the diagnosis of echinococcus granulosus (9). Cyst aspiration-fluid polymerase chain reaction may also be useful for diagnosis.

Treatment modalities include puncture aspiration injection reaspiration, which is reserved for uncomplicated cysts that do not have daughter cysts (e.g. WHO stage CE1 and CE3a) (3). Percutaneous treatment is associated with a risk of anaphylaxis. Modified catheterization techniques are used to remove the entire endocyst and daughter cysts from the cyst cavity using large bore catheters and cutting devices together with an aspiration apparatus. Drug therapy may be used for definitive or adjunctive therapy. Albendazole is the primary antiparasitic agent for the treatment of Echinococcus granulosus. Surgery is the treatment of choice for complicated cysts or for cysts with many daughter vesicles (e.g. WHO stage CE2 and CE3b). Due to its high antigenic nature, the toxic fluid of hydatid cyst cause anaphylaxis and recurrence. Hence, cysts should be removed intact and if spillage occurs, 15 to 20% hypertonic saline wash may be used. The WHO recommends postoperative chemotherapy with albendazole for 1 month or mebendazole for 3 months if spillage occurs (3). A high index of suspicion must be kept for this disease because the incidence of pelvic hydatid cyst is very low and it mimics ovarian malignancy or other ovarian tumors.

Our patient presented with cysts belonging to stages C2 and C3b for which the preferred treatment is albendazole followed by surgery, which was implemented for this patient (3). The patient was healthy at the time of writing this case report having completed a 3-month course of albendazole postoperatively.

## Figures and Tables

**Table 1:**
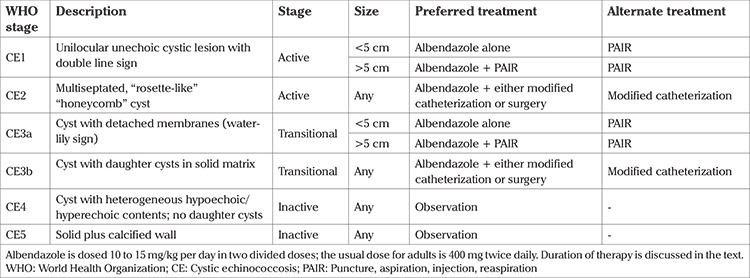
World Health Organization classification of cystic echinococcosis and treatment stratified by cyst stage

**Figure 1:**
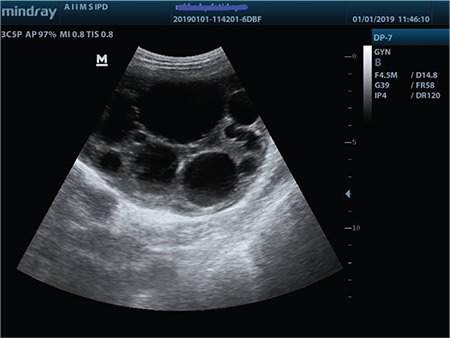
Ultrasonography pelvis showing multiseptated cystic lesion with multiple small cysts within both adnexae

**Figure 2:**
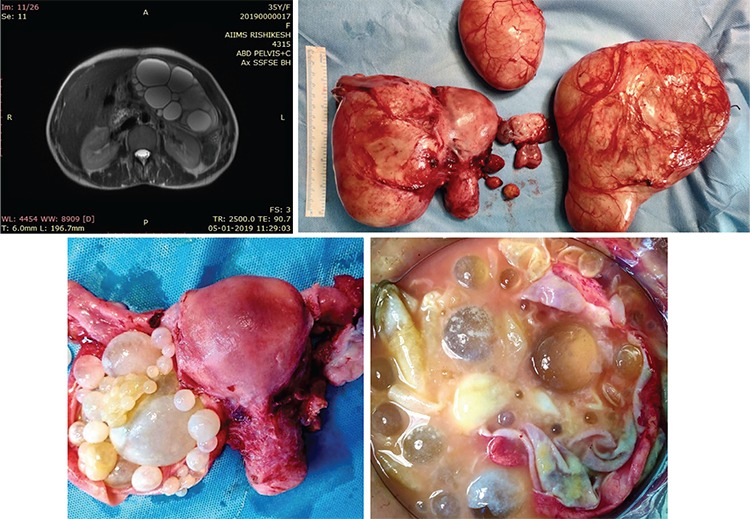
T2-weighted magnetic resonance imaging image showing smooth walled clusters of fluid signal cystic lesion in the pelvis with clusters of multiple tiny fluid signal cysts. Intra-operative finding showing multiple fluid filled cysts in the ovary and omentum

**Figure 3:**
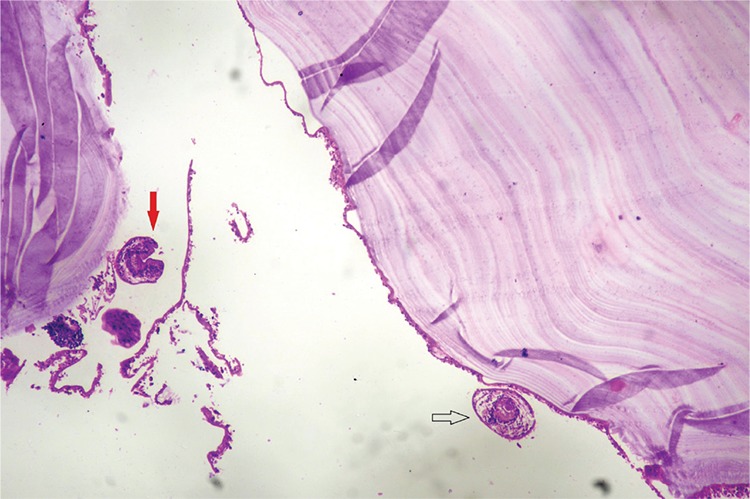
Hematoxylin and eosin (100x), section shows lamellated membranes of Echinococcus granulosus. Also seen are brooding daughter cysts (black arrow) and the scolex of the parasite (red arrow)
